# A clinician’s guide for developing a prediction model: a case study using real-world data of patients with castration-resistant prostate cancer

**DOI:** 10.1007/s00432-020-03286-8

**Published:** 2020-06-17

**Authors:** Kevin M. Veen, Isabel B. de Angst, Mostafa M. Mokhles, Hans M. Westgeest, Malou Kuppen, Carin A. Uyl-de Groot, Winald R. Gerritsen, Paul J. M. Kil, Johanna J. M. Takkenberg

**Affiliations:** 1 Department of Cardio-Thoracic Surgery, Erasmus Medical Center, Rotterdam, The Netherlands; 2 Department of Urology, Elisabeth-Tweesteden Hospital, Tilburg, The Netherlands; 3 Department of Internal Medicine, Amphia Hospital, Breda, The Netherlands; 4 Institute for Medical Technology Assessment, Erasmus School of Health Policy and Management, Erasmus University, Rotterdam, The Netherlands; 5 Department of Medical Oncology, Radboud University Medical Center, Nijmegen, The Netherlands

**Keywords:** Decision-making, Prediction modeling, Castration-resistant prostate cancer, Cox proportional hazard model

## Abstract

**Purpose:**

With the increasing interest in treatment decision-making based on risk prediction models, it is essential for clinicians to understand the steps in developing and interpreting such models.

**Methods:**

A retrospective registry of 20 Dutch hospitals with data on patients treated for castration-resistant prostate cancer was used to guide clinicians through the steps of developing a prediction model. The model of choice was the Cox proportional hazard model.

**Results:**

Using the exemplary dataset several essential steps in prediction modelling are discussed including: coding of predictors, missing values, interaction, model specification and performance. An advanced method for appropriate selection of main effects, e.g. Least Absolute Shrinkage and Selection Operator (LASSO) regression, is described. Furthermore, the assumptions of Cox proportional hazard model are discussed, and how to handle violations of the proportional hazard assumption using time-varying coefficients.

**Conclusion:**

This study provides a comprehensive detailed guide to bridge the gap between the statistician and clinician, based on a large dataset of real-world patients treated for castration-resistant prostate cancer.

**Electronic supplementary material:**

The online version of this article (10.1007/s00432-020-03286-8) contains supplementary material, which is available to authorized users.

## Introduction

As an urologist or oncologist it is not rare to encounter a 77 year old prostate cancer patient treated with androgen deprivation therapy, whose PSA rises consecutively at castrate serum levels of testosterone and who develops new bone lesions on imaging studies. According to the European Association of Urology guidelines, this patient meets the criteria for metastatic Castration-Resistant Prostate Cancer (CRPC) (Cornford et al. 2017). The patient has a medical history of chronic obstructive pulmonary disease (COPD) and diabetes mellitus. He has no prostate cancer related symptoms but due to his comorbidities he has a performance status of 1. We have previously shown that based on these factors Dutch clinicians are more likely to opt for watchful waiting or hormone targeted drugs, instead of docetaxel/prednisolone or radium-223 (Angst et al. 2019). In absence of clear recommendations for a preferred treatment option and sequence, clinicians may benefit from support of a clinical prediction model that is able to predict survival per treatment option based on patients’ clinical baseline characteristics.

Recently, a significant amount of work has been published concerning risk prediction in prostate cancer (Kearns and Lin 2017). Risk prediction models evolved to indispensable tools to aid clinicians in making evidence-based decisions. In the urology field clinical risk prediction models for different disease states of prostate cancer exist, to predict for example the probability of biopsy-detectable aggressive prostate cancer, lymph node involvement, or overall survival (OS) in first-line chemotherapy. Nevertheless, despite existing general guidelines for reporting of a multivariable prediction model for individual prognosis or diagnosis (Collins et al. 2015), the process of developing and validating such models is still shrouded in mystery for most clinicians. The aim of this paper is to provide a comprehensive detailed guide to help clinicians understand the (sometimes complex) steps in developing a useful prediction model for CRPC patients, based on a real-life case, using a retrospective dataset of real-world patients treated for CRPC. We aim to both assist the clinician in understanding the development of a prediction model and to support the clinician in recognizing common shortcomings in existing prediction models. Of course, it is of highly importance to involve a statistician in the preparatory phase as well as constructing and validating the model.

## Methodology

### Research question and statistical model choice

First and foremost, one needs to formulate a clear research question. Additionally, before delving into the process of developing a prediction model it should first be checked if a similar model exists. In this case it may sometimes be more appropriate to update or adapt these previous models. In this study we aimed to develop a model to predict mortality in patients with CRPC treated in first-line with either abiraterone, enzalutamide, docetaxel, watchful waiting (defined as best supportive care using systemic treatment without proven life prolonging benefits, such as anti-androgens and ketoconazole) or radium-223, with the goal to use the model for treatment decision-making and to incorporate the model into a decision aid. Based on the type of outcome an appropriate model should be chosen, because different models should be used for different types of data (Supplementary Table 1). In our case we are dealing with survival data. Hence, a non-parametric Cox proportional hazard model was chosen. It should be noted that for very long-term predictions a parametric model (e.g. Weibull) may be preferred, since these provide more stable predictions at the end of follow up (Carroll 2003). A summary of all considerations in model development is presented in Table 1.

**Table 1:** Summary of considering in prediction modelling. [adapted from original version of Steyerberg et al. (2008)]

Step	Specific issues	CAPRI-dataset
General considerations		
Research question	Aim: predictors/prediction	Prediction
Intended application	Clinical practice/research	Clinical practice
Outcome	Clinically relevant	Mortality
Predictors	Reliable measurement Comprehensiveness	Oncological clinical work-up and literature; extensive set of candidate predictors
Study design	Retrospective/prospective? Cohort; case–control	Registry study: retrospective cohort
Statistical model	Appropriate for research question and outcome	Non-parametric cox proportional hazard
Sample size	Sufficient for aim?	3588 patients; 2335 events
5 modelling steps		
Data inspection	Data distribution Missing values Correlation between predictors	Table 2 (baseline table) Multiple imputation Using Pearson’s R or Spearman’s rho
Coding of predictors	Continuous predictors	Extensive checks of transformations for continues predictors
	Combining categorical predictors	Comorbidity score was collapsed to three categories instead of eight
	Combining predictors with similar effects	Pain and opioid use
Model specification	Appropriate selection of main effects	LASSO regression
	Assessment of assumptions	Additivity checked with interaction terms, interaction with treatment was checked, three interaction terms included Proportional hazard assumption checked—> relaxed by time varying coefficients
Model performance	Appropriate measures	Discrimination
Model validation	Internal validation External validation	Bootstrap and k-fold cross-validation No external dataset was available

### Data inspection

In our case we used a retrospective registry called the CAstration-resistant Prostate cancer RegIstry (CAPRI), which is an investigator-initiated, observational multi-center registry in 20 hospitals in the Netherlands. In the subset of the data we used, with first line treatment only, 3588 patients and 2335 deaths were recorded (Westgeest et al. 2018). The patients were treated according to clinical practice with a variety of first-line treatments including abiraterone, enzalutamide, docetaxel, or watchful waiting. Radium-223 was excluded from analyses due to the fact that only ten patients received Radium-223 as first line treatment in this dataset. Baseline variables are presented in Table 2. Furthermore, this dataset contained sixteen potential predictors. In general, it is recommended to have at least ten events (deaths in our case) to investigate one predictor. If a predictor has multiple categories you need 10*(number of categories − 1) events for that predictor.

**Table 2:** Baseline characteristics of patients with CRPC treated with abiraterone, enzalutamide, docetaxel or watchful waiting

Treatment	Abiraterone	Enzalutamide	Docetaxel	Watchful waiting
*n*	249	184	1006	2149
Anti-androgens before CRPC (%)	114 (46.0)	81 (44.0)	397 (39.5)	788 (36.8)
Comorbidity score (%)				
0	168 (67.5)	107 (58.2)	703 (70.0)	1227 (57.1)
1	43 (17.3)	38 (20.7)	185 (18.4)	496 (23.1)
2	24 (9.6)	23 (12.5)	80 (8.0)	252 (11.7)
3	6 (2.4)	6 (3.3)	22 (2.2)	86 (4.0)
4	5 (2.0)	4 (2.2)	8 (0.8)	46 (2.1)
5	0 (0.0)	2 (1.1)	3 (0.3)	13 (0.6)
6	3 (1.2)	2 (1.1)	4 (0.4)	17 (0.8)
7	0 (0.0)	1 (0.5)	0 (0.0)	5 (0.2)
8	0 (0.0)	1 (0.5)	0 (0.0)	5 (0.2)
Bone metastases (%)	142 (87.7)	103 (87.3)	703 (91.1)	929 (81.7)
Lymph node metastases (%)	66 (80.5)	41 (83.7)	373 (82.5)	507 (76.6)
Visceral metastases (%)	8 (16.7)	8 (24.2)	57 (21.7)	52 (16.1)
WHO (%)				
1	37 (40.2)	26 (43.3)	222 (42.0)	360 (47.1)
2	41 (44.6)	21 (35.0)	245 (46.3)	317 (41.5)
3	14 (15.2)	13 (21.7)	62 (11.7)	87 (11.4)
Pain (%)	47 (42.0)	28 (37.8)	317 (49.2)	323 (31.0)
Opioid use (%)	22 (32.8)	9 (24.3)	120 (29.3)	113 (22.7)
Gleason > 7 (%)	143 (67.8)	105 (65.2)	591 (65.9)	998 (55.5)
Time to castration (median [range])	11.17 [1.4, 192]	13.34 [1, 196]	10.12 [0.2, 172.7]	20.47 [0.3, 248.4]
Age (median [range])	76.00 [46, 95]	77.00 [50, 94]	70.00 [46, 93]	78.00 [49, 99]
Weight (median [range])	83.00 [52, 120]	86.00 [60, 120]	84.50 [48, 150]	81.00 [44, 118]
Hemoglobulin (median [range])	8.00 [5.1, 9.6]	8.00 [4.7, 10.3]	8.00 [4.3, 10.2]	8.10 [3.9, 10.5]
Platelets (median [range])	234.00 [37, 569]	228.50 [54, 473]	243.00 [0.4, 749]	233.00 [0.3, 714]
Lactate dehydrogenase (median [range])	218.00 [72, 3179]	216.00 [98, 730]	232.00 [21, 4100]	218.00 [79, 4329]
Alkaline phosphatase(median [range])	122.00 [41, 1673]	109.00 [38, 1263]	136.00 [34.8, 3457]	93.00 [21, 4315]
PSA (median [range])	34.00 [0.1, 8730]	24.40 [0.1, 4150]	40.00 [0.0, 8700]	9.70 [0.1, 4034]

### Missing values and coding of predictors

In an ideal world the predictors in a dataset are all clinically relevant (Cornford et al. 20172), comprehensible (Angst et al. 2019), measured reliably (Kearns and Lin 2017), without missing data (Collins et al. 2015), and not correlated with each other (Carroll 2003). Unfortunately, datasets fulfilling all these criteria are the exception rather than the rule. Regarding the first three criteria it is recommended that clinician’s perspectives are taken into account. Several authors mentioned to perform systematic reviews in order to find suitable candidate predictors (Steyerberg 2008). In the sections below we will address the latter two criteria (missing values and correlation between predictors). Additionally, we will give special attention on how to handle continuous predictors (e.g. age and hemoglobin).

### Missing values

Various approaches are described to handle missing data, each with its own limitations and benefits (Papageorgiou et al. 2018). In our case we used multiple imputation using the MICE statistical package of R (Buuren and Groothuis-Oudshoorn 2011). “Imputation” in the context of missing baseline variables basically means that missing values are predicted upon other baseline values and/or outcome. Alike almost every statistical manipulation, certain assumptions must be made about the missing data, especially the mechanism of missing data (missing completely at random, missing at random, missing not at random) should be addressed (Papageorgiou et al. 2018). Following the latest consensus we incorporated the outcome in the imputation model using the Nelson-Aalen estimator, a non-parametric estimator of the cumulative hazard rate function (Moons et al. 2006). Using multiple imputation one creates multiple datasets in which the missing values are imputed, resulting in multiple completed datasets. The formal rules state that the analyses need to be conducted on all datasets separately and the obtained estimated must be pooled thereafter (Rubin 2004). Nevertheless, in case of a few missing values some authors proposed to develop the model on one dataset and test the model on the other datasets (Steyerberg 2008). Controversy remains on the cut-off of how much missing values is “too much” missing (Papageorgiou et al. 2018).

### Correlation between predictors

In medicine many variables roughly describe the same phenomena and are therefore correlated with each other. One should avoid putting highly correlated variables in the same model. Firstly, the aim of a prediction model is to be as simple as possible, and incorporating similar variables is considered redundant. Secondly, in case of correlated variables a phenomena called “multicollinearity” can occur, characterized by extremely high/low estimates or standard errors (Multicollinearity 2020). Therefore, it is advisable to investigate all the correlations between the predictors by means of Pearson’s R or Spearman’s rho, and high correlation should be addressed. This can either be done by excluding one of the two correlated variable or recoding the variables into one new variable. In our case the variables “pain” and “opioid use” were correlated (Spearman’s rho: 0.36). Clinically this makes perfect sense, as opioids are prescribed when a patient is in pain. We recoded opioid and pain in several variables and a combined variable consisting out of 3 categories proved to be the best predictor (Supplementary Table 3).

### Continuous predictors

**Fig. 1:**
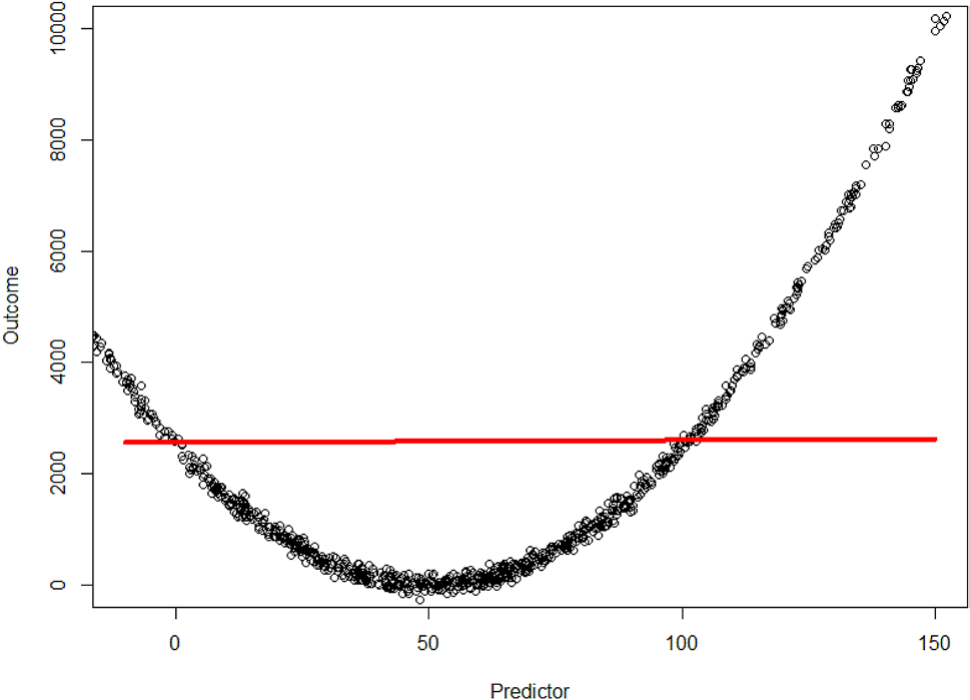
Example of a continuous outcome (*y*-axis) and continuous predictor (*x*-axis). As is shown: with the assumption the relation is linear the model (red line) does not fit the observed data well (black dots)

**Fig. 2:**
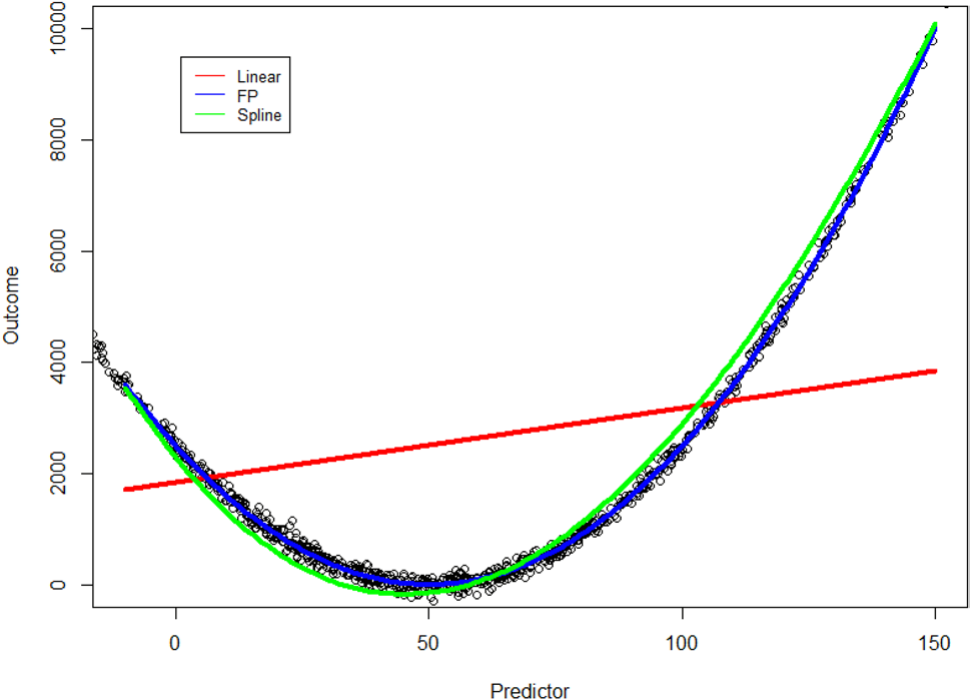
Example of relaxation of the linear assumed association (red line) of a continuous outcome and predictor. This can be done either with natural splines (green line) or fractional polynomials (FP) (blue line). Using splines the data is divided in separate sections, and each section has its own estimate of the line. Using fractional polynomials the relationship is described as multiple polynomials, which can produce a very flexible line

Continuous predictors are variables that can take an infinite number of values (e.g. age and lactate dehydrogenase), and contain a lot of information. Hence, simply dichotomizing continuous predictors is paired with significant information loss (Royston et al. 2006). Nevertheless, incorporating continuous predictors into a statistical model comes along with the assumption the continuous predictors is associated with the outcome in a linear way. While a linear association can also be applied for some non-linear associations, this may not always be the case (Fig. 1). Thus, we recommend firstly to explore the association of the continuous predictor with the outcome in a univariable model. In order to explore the best fitting association with the outcome and a continuous predictor one can use: transformation (like logarithmic transformation), categorization, splines and fractional polynomials, as is explained in Table 3 and Fig. 2 (Steyerberg 2008).

**Table 3:** Performance of a linear model by adding flexibility to assumed linear association with the outcome

Variable	*R*-squared*
Predictor linear	0.00938
Predictor with splines with one knot	0.9853
Predictor with fractional polynomial	0.9992

*R-squared is measure of how close the model fits the data, 1 indicates the model explains all the variability of the data, whereas with 0 the model does not explain any variability. For other types of models similar measurements are available

### Interaction

Let us consider two predictors. Separately, they have no association with the outcome, however, when they are both present, a significant association with the outcome is observed (or vice versa). Such a phenomena is called “interaction” (Steyerberg 2008). For example these interactions are quite common in gene studies: Only when gene X and gene Y are turned on a certain chemical reaction will start. When either one of the genes is turned off, the reaction will not begin. Naturally, these interactions can also be present in epidemiology studies. However, especially when one considers many predictors, constructing interaction terms can be an overwhelming task. There are so many possibilities one cannot see the wood for the trees. In this case it is advisable to avert to the clinicians and a priori select a number of possible interactions, which make clinical sense. In our study, we tested the interaction term “watchful waiting” and “opioid use or pain”, which turned out to be highly significant. This corresponds to the clinic; a patient with watchful waiting and opioid use or pain indicates a palliative setting, in which the patient is expected to die soon. Hence, watchful waiting and opioid use together have a stronger association with the outcome than watchful waiting and opioid use separately.

### Model specification

As mentioned earlier, the first step of predictor selection should be together with subject-specific experts. Predictor selection is arguably the hardest part of model building (Ratner 2010). Multiple methods exist to address the selection process of the a priori selected set of predictors. The most widely used methods include stepwise selection and best subset regression, and these are previously described (Miller 2002; Harrell 2015). In our case we had a lot of variables due to the interaction terms and non-linear continuous predictors. One always wants the most parsimonious model and does not want to exceed the one predictor per ten events rule of thumb. Therefore, it is reasonable to drop predictors that do not add much to the performance of the model. We employed a lesser known selection method using Least Absolute Shrinkage and Selection Operator (LASSO) regression (Tibshirani 1996). This is a penalized machine learning technique that shrinks the estimate of unimportant predictors to zero (Supplementary Fig. 1). An estimate of zero equals no association with the outcome and, therefore a predictor is excluded. This method also can handle correlation within predictors to some extent, as the algorithm will “see” that in case of high correlation of predictor A and B, shrinking predictor B to zero will not influence performance of the model (Tibshirani 1996). Nevertheless, an algorithm cannot judge which predictor is more comprehensible or measured reliably. Therefore, one should never skip the step of looking for correlations between predictors. A package to run LASSO regression in R is the “glmnet” package (Friedman et al. 2010), with an elaborate vignette to code this in R (Hastie and Qian 2016). However, in our case we had multiple polynomials describing the relation of a continuous predictor with the outcome (see “Continuous predictors”). One wants either include all the polynomials in the model or none at all. Hence, we need to “tell” the LASSO algorithm they belong together as a group. The statistical R package “grpreg” has implemented such a function (Breheny and Huang 2015).

We opted for a two-step approach. Firstly, we ran the LASSO regression and thereafter we incorporated all the non-zero predictors in a Cox-model. The final model is shown in Table 4.

**Table 4:** Final Cox model for predicting mortality in patients with CRPC

Characteristic	Hazard ratio (95% CI)	*P* value
Age	1.07 (1.04–1.09)	< 0.001
Anti-androgens before CRPC	0.87 (0.8–0.95)	0.001
Bone metastases	1.16 (1.03–1.32)	0.016
AF polynomial 1^1^	1.02 (0.9–1.16)	0.75
AF polynomial 2^2^	0.75 (0.57–0.99)	0.044
Enzalutamide vs abiraterone	1.17 (0.64–2.15)	0.60
Docetaxel vs abiraterone	1.85 (1.23–2.77)	0.003
Watchful waiting vs abiraterone	0.45 (0.31–0.67)	< 0.001
Time to start castration spline 1 h _for <10 months_	0.2 (0.1–0.39)	< 0.001
Time to start castration spline 2 h _for <10 months_	0.19 (0.13–0.26)	< 0.001
Time to start castration spline 1 h _for>10 months_	1.45 (0.75–2.8)	0.27
Time to start castration spline 2 h _for>10 months_	0.71 (0.51–1)	0.048
WHO _HR for <10 months_	1.64 (1.44–1.87)	< 0.001
WHO _HR for >10 months_	1.07 (0.99–1.15)	0.11
PSA polynomial 1^3^ h _for <10 months_	1.34 (1.15–1.56)	< 0.001
PSA polynomial 1^3^ h _for >10 months_	1.02 (0.88–1.17)	0.82
PSA polynomial 2^4^ h _for <10 months_	1.27 (1.16–1.4)	< 0.001
PSA polynomial 2^4^ h _for >10 months_	1.11 (1.01–1.21)	0.023
HB _HR for <10 months_	0.82 (0.76–0.89)	< 0.001
HB _HR for>10 months_	0.92 (0.87–0.97)	0.003
Platelets polynomial 1^5^_HR for <10 months_	0.97 (0.95–0.99)	0.001
Platelets polynomial 1^5^_HR for >10 months_	1.01 (0.99–1.02)	0.42
Platelets polynomial 2^6^_HR for <10 months_	1 (1–1.01)	0.001
Platelets polynomial 2^6^_HR for <10 months_	1 (1–1)	0.46
LDH _HR for <10 months_	1.66 (1.42–1.94)	< 0.001
LDH _HR for>10 months_	1.09 (0.96–1.23)	0.18
Opioid or pain vs none _HR for <10 months_	1.09 (0.97–1.22)	0.16
Opioid or pain vs none _HR for >10 months_	1.02 (0.94–1.09)	0.67
Age*Enzalutamide vs abiraterone^7^	0.94 (0.9–0.97)	0.001
Age*Docetaxel vs abiraterone^7^	0.96 (0.93–0.99)	0.003
Age*Watchful waiting vs abiraterone^7^	0.99 (0.96–1.01)	0.25
Log(PSA)*Enzalutamide vs abiraterone^7^	1.08 (0.92–1.26)	0.35
Log(PSA)*Docetaxel vs abiraterone^7^	0.91 (0.83–1)	0.057
Log(PSA)*Watchful waiting vs abiraterone^7^	1.23 (1.12–1.35)	< 0.001

The model contains fractional polynomials and splines to address non-linear associations of a continues variable with the outcome and a stepwise time-varying coefficient function; e.g. some covariates have a hazard ratio for below ten months of follow-up and above ten months of follow-up

1:(AF/100)^−2), 2: (AF/100)^−1, 3: PSA^−1, 4: log(PSA), 5: Platelets*1, 6: Platelets * log(Platelets), 7: interaction term

### Assessment of assumptions

**Fig. 3:**
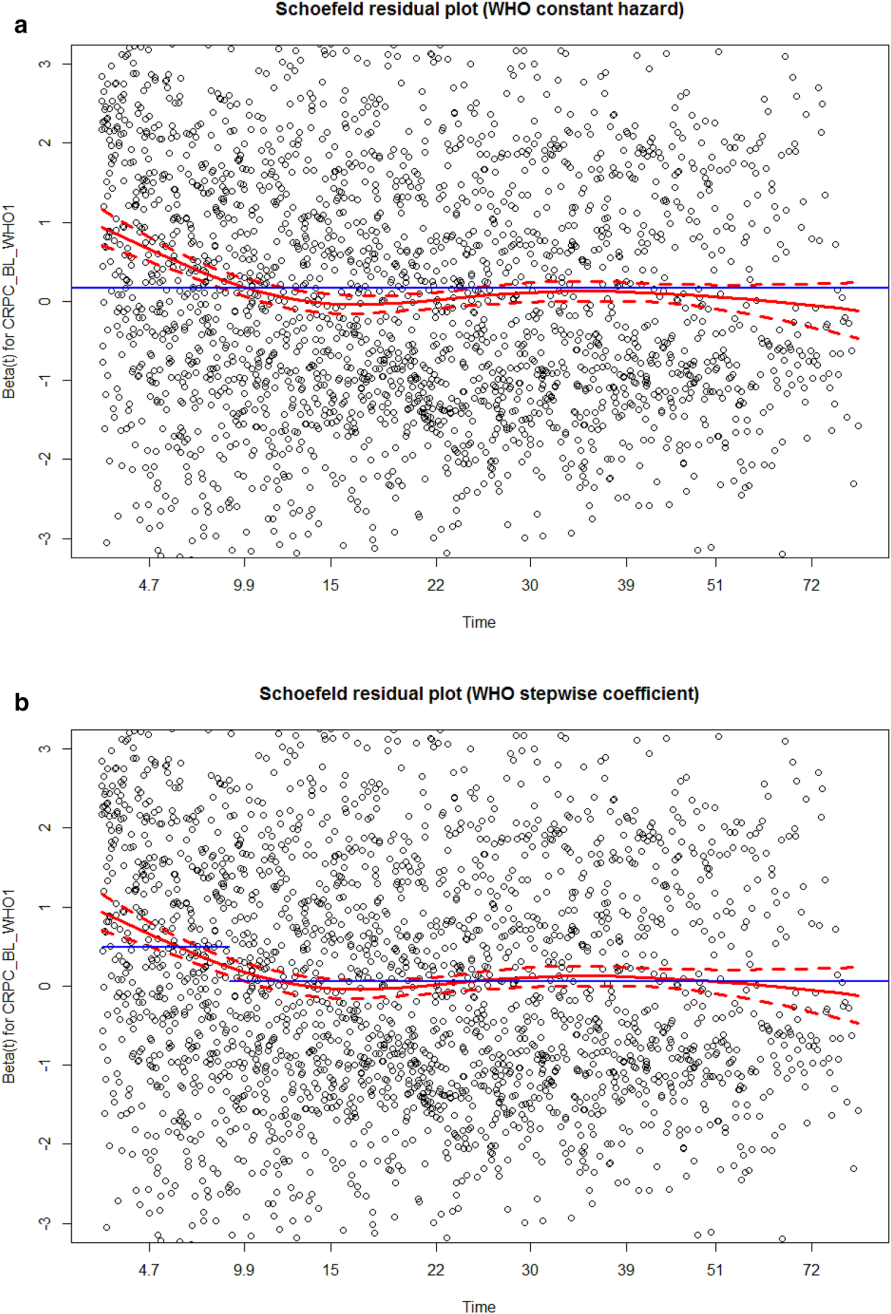
**a** Example of a Schoenfeld residuals plot in order to check the proportional hazard assumption. When the hazard of WHO is assumed constant over time (blue line in part **a**), the assumption is violated, especially in the first ten months the blue line deviates from the red line. In part **b** we have two coefficients for WHO, one for the first ten months and one for more than ten months. Proportional hazards assumption is not violated anymore

Every statistical model comes along with certain assumptions (Freedman 2009). If these assumptions are not met, the model is not or less valid (Freedman 2009). Each model family has its own specific assumptions. A key assumption in the cox model we used is the proportional hazard (PH) assumption. This basically means that ratio of hazards (the output of a Cox model) is constant over time. Two approaches are commonly used to test whether this assumption is violated: plotting Kaplan–Meier curves or plotting the residuals. Both methods are implemented in most statistical programs or packages. The Schoenfeld residuals should be used to test the PH assumption. Schoenfeld residuals represent the difference between the observed covariate and the expected given the risk set at that time. If one draws an average line through the residuals, this line should be straight (Schoenfeld 1982). A formal test has also been developed (Schoenfeld *F* test) (Grambsch and Therneau 1994). In our model certain variables did not meet the PH assumption. Fortunately, this is not the end of the world. One can avert to parametric models, since some of these models do not rely on the PH assumption, however you need to start all over again. Another approach is to use an extension of the Cox model called time-varying coefficients, not to be confused with time-varying covariates (Hastie and Tibshirani 1993; Fisher and Lin 1999). Time-varying coefficients can be applied if the effect of a predictor is not constant over time, or in other words if the PH assumption is violated. In our case the effect predictor WHO performance status was not constant over time. As is shown in the Schoenfeld residual plot the effect of the performance status was higher in the first months compared to later in follow-up (Fig. 3a). Therefore, we decided to use a stepwise time-varying coefficient function; we made a separate hazard ratio for the first ten months and for the following months thereafter. As presented in Fig. 3b, the PH assumption was not violated anymore. A vignette to implement time-varying coefficients in R has been published previously (Therneau et al. 2013).

### Model performance

Two related terms are important in model performance: discrimination and calibration (Alba et al. 2017). Discrimination describes how well a model discriminates a high risk patient from a low risk patient or, in other words: Does the model estimate higher probabilities for patients that have an event compared to patients that do not have an event? Discrimination of binary outcomes is measured with the c-statistic or with ROC-curves (Pencina and D'Agostino 2015). In our study, the overall c-statistic of the model was 0.74, which indicates a good discrimination of the model. Calibration or goodness-of-fit conveys to which extent the predicted probability agrees with the observed probability. For example a high risk patient had a sevenfold higher probability of an event compared to a low risk patient and predicted risks are 7% vs 1%. The observed probabilities of a high risk patient and a low risk patient were 70% vs 10%. In this case discrimination is satisfactory, as the model discriminates well between a high and low risk patient. Nevertheless, calibration is extremely off; the observed risks are not even close to the predicted risks. Several methods exist to assess calibration and are described previously (Calster et al. 2016).

### Model validation

Testing model performance on the dataset on which is developed is most of the time overly optimistic (Babyak 2004). After all, the model “learned” the estimates out of the correlations/associations derived from that specific dataset. To assess the possibly overly optimistic performance a statistical model should be validated. Preferably, this should be done internally and externally. During internal validation the model is validated with the original dataset. Historically, this is done by randomly splitting the original dataset into two datasets. One training dataset and one validation dataset. Nevertheless, this approach is not recommended, because this inherently implies one cannot train the model on all the patients. In small datasets the amount of data is reduced, possibly leading to overfitting, and in very large datasets randomly splitting results in very comparable datasets. Therefore, we recommend to employ either bootstrapping techniques or *k*-fold cross validation. Using k-cross validation one uses the whole dataset as training dataset for the model, and thereafter splits the dataset in *k* groups (usually ten groups). One group is the validation set and the others are the training sets. This process is repeated *k* times with each a different group for the validation set (Supplementary Fig. 3) (Harrell 2015). Using bootstrapping the model is also trained on the whole dataset and thereafter random samples are drawn from the original data. Herein a patient can be drawn multiple times and the drawn sample is usually of the same size of the original dataset (Supplementary Fig. 4) (Efron and Tibshirani 1994).

Notwithstanding, the ultimate test for a model is external validation. This means that the performance of the model is still satisfactory if it is tested on a different dataset. For example this dataset could be derived from another center, or geographical area. A model that calibrates poorly on external data can be recalibrated, whereas a model that discriminates poorly cannot. In this case a new model is required (Su et al. 2018).

There is another highly important form of validity called “face validity”. Yet, again the expert clinician comes into play here, as there are no formal ways to test face validity. Face validity says something about whether the test or model measures what it is supposed to measure. For instance face validity may be impaired when key predictors are not included in the model because they were not collected. Or when the dataset is old and does not represent clinical practice anymore. In our case, the patients in the CAPRI dataset were included from January 1, 2010 until December 31*,* 2017*.* Our aim was to develop a model to predict mortality in patients with CRPC treated with either abiraterone, enzalutamide, docetaxel, or watchful waiting in first line, to support adequate decision making. However, due to the retrospective nature of this dataset, strong selection bias is present for treatment, especially since abiraterone and enzalutamide were not available as first-line treatment in the Netherlands from 2010–2013. So patients that were eligible for those treatments, received watchful waiting or docetaxel in this period. Of course, a multivariable model will adjust to some extend for this, and one can include intervention year as covariate to assess/and adjust for this phenomena. However, for future predictions, intervention year as covariate implies that a certain trend will continue in the future. This does not make (clinical) sense at all. Hence, this model failed the face validity.

## Conclusion

Risk prediction is becoming increasingly more important in medical practice. In this article, we discuss several steps in developing a prediction model including missing data, predictor encoding and selection using LASSO, testing model assumptions, performance and validation, using an example from uro-oncology. Prediction model development is not a futile task and both the input of the clinician and statistician are essential. This article may be used to bridge the gap between the two disciplines.

## Electronic supplementary material

Below is the link to the electronic supplementary material.

Supplementary file1 (DOCX 1827 kb)
