# Supplementary material for: A clinician’s guide for developing a prediction model: a case study using real-world data of patients with castration-resistant prostate cancer
Source: J Cancer Res Clin Oncol. 2020 Jun 17;146(8):2067–75. doi: 10.1007/s00432-020-03286-8 (PMC7324416; doi:10.1007/s00432-020-03286-8)

Contents

[Supplementary Table 1 2](#_Toc11246467)

[Supplentary Table 2 3](#_Toc11246468)

[Supplementary figure 1 4](#_Toc11246469)

[Supplementary Figure 2 5](#_Toc11246470)

[Supplementary figure 3 6](#_Toc11246471)

Supplementary Table 1: Types of data and their associated models

| **Type of data** | **Example** | **Regression model** |
| --- | --- | --- |
| Continuous | Blood pressure, age | Linear regression |
| Discrete | Yes/no variables | Logistic regression |
| Count data (special case of continuous data) | Hospital stay | Poisson regression  Negative binomial regression |
| Ordinal data | WHO class | Ordinal regression |
| Survival data | Mortality | Cox regression (non-parametric)  Accelerated time failure models (parametric) |

Supplementary Table 2: The variables opioid and pain were highly correlated. Hence, these variables was combined in several ways and it was tested which variable had the best prediction. 1 is if the characteristics is present and 0 when not.

AIC =Akaike information criterion and BIC =Bayesian information criterion, both are comparative measurements of the fit of a model, penalized for the number of fitted covariates. A lower AIC and BIC indicate a better model.

| Name recoded variable | Recoding scheme | AIC | BIC |
| --- | --- | --- | --- |
| Opioid and pain | If opioid = 1 AND pain = 1 -> Opioid and pain = 1  Else: Opioid and pain = 0 | 35954.57 | 35960.37 |
| Opioid or pain | If opioid=1 OR pain = 1 -> Opioid or pain = 1  Else: Opioid or pain = 0 | 35962.20 | 35968.00 |
| Ordered opioid and pain_3 (3 levels) | If opioid =1 OR pain = 1 -> Ordered opioid and pain_3 = 1  If opioid =1 AND pain = 1 -> Ordered opioid and pain_3 = 2  Else: Ordered opioid and pain_3 = 0 | 35910.92 | 3596.72 |
| Ordered opioid and pain_4 (4 levels) | If pain = 1 -> Ordered opioid and pain_4 = 1  If opioid = 1 -> Ordered opioid and pain_4 = 2  If opioid =1 AND pain = 1 -> Ordered opioid and pain_4 = 3  Else: Ordered opioid and pain_4 = 0 | 35911.34 | 35922.93 |

Supplementary figure 1: Shrinkage of predictors to zero using LASSO regression


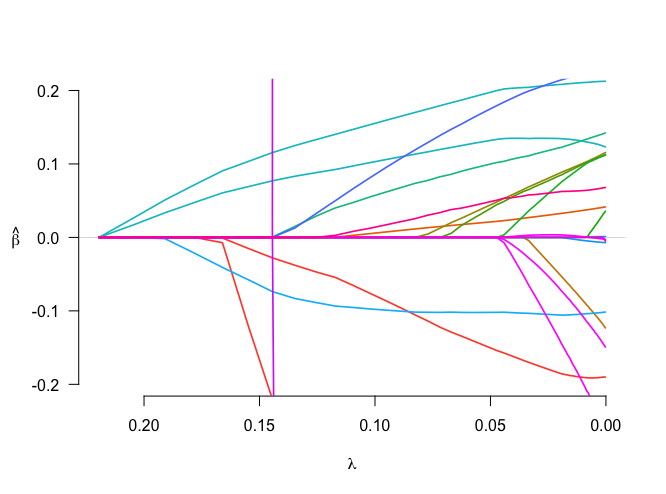


Supplementary Figure 2: Schematic of k-fold cross validation in which k=4.


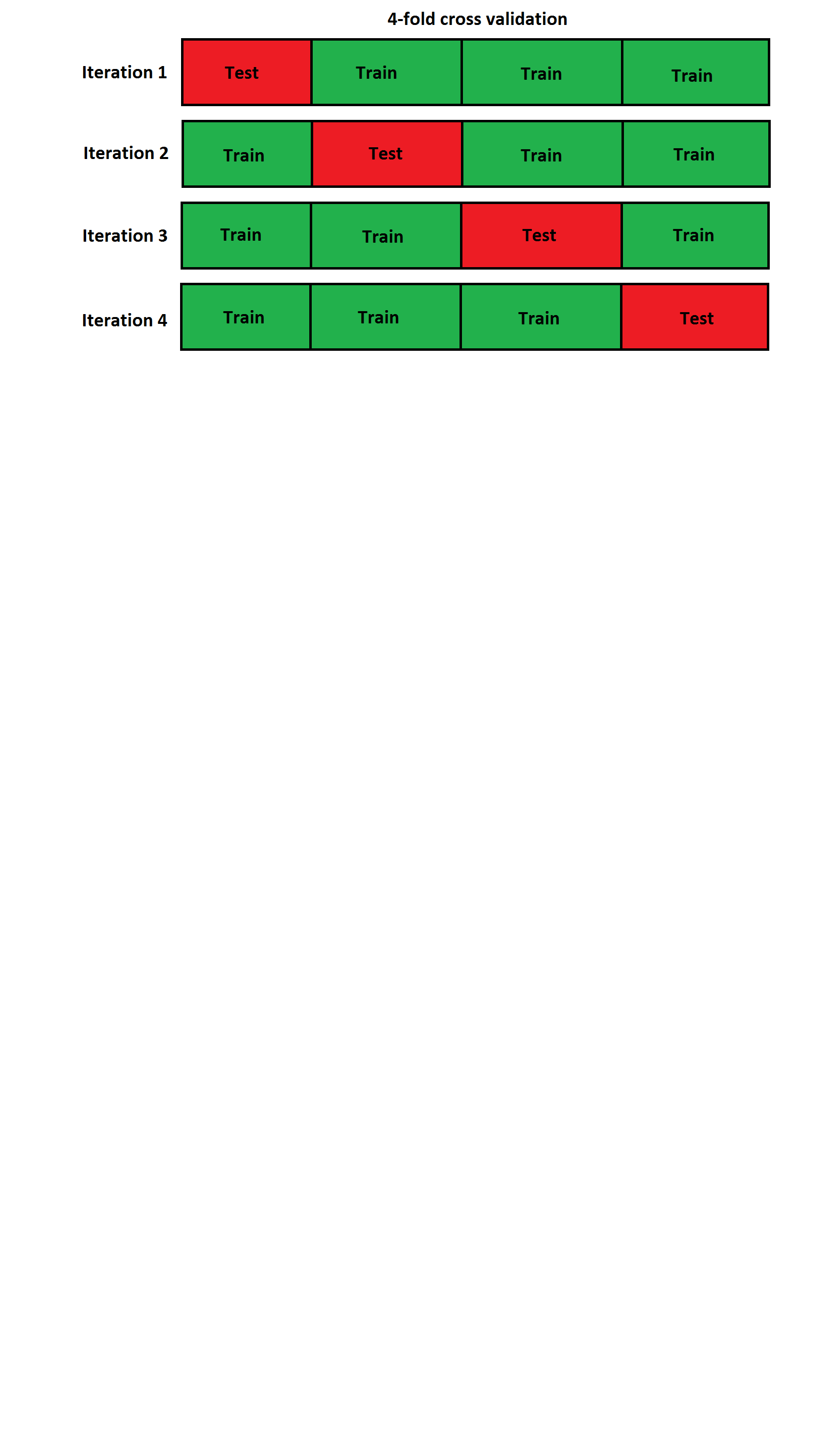


Supplementary figure 3: Schematic of bootstrapping. The general idea behind bootstrapping is that of the original sample several bootstrap samples can be drawn with the same sample size as the original sample. In the bootstrap sample *replacement* is possible (e.g. the same subject can be drawn multiple times and at every step every subject has equal probability to be selected). Bootstrapping can be used to test model performance.


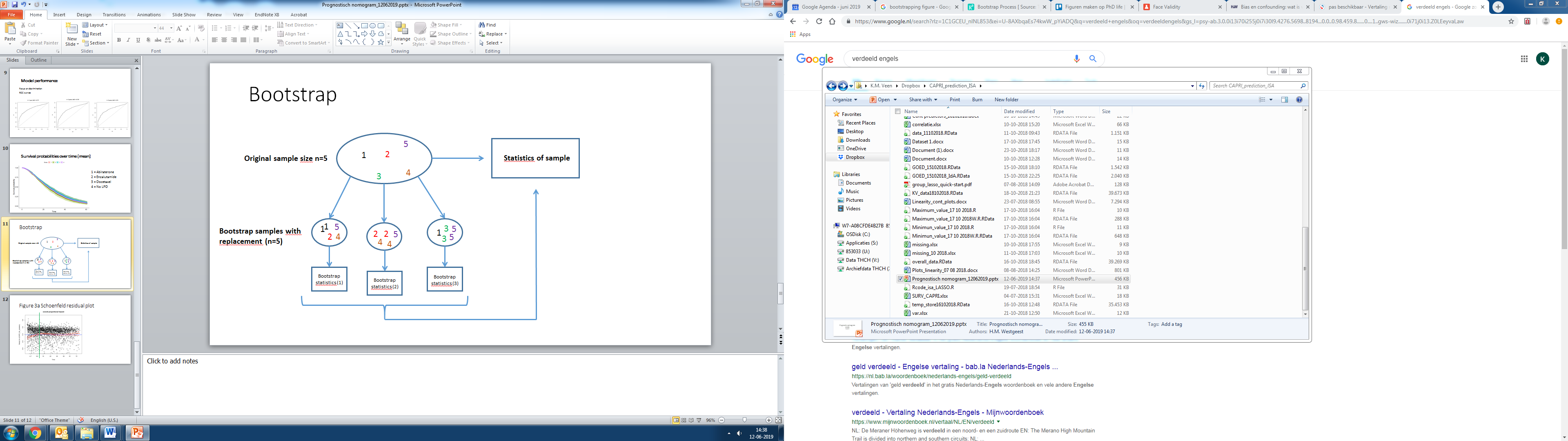

Supplement: Supplementary file 1 — Supplementary file1 (DOCX 1827 kb) [file 432_2020_3286_MOESM1_ESM.docx]
